# The Sexual and Mating System of the Shrimp *Odontonia katoi* (Palaemonidae, Pontoniinae), a Symbiotic Guest of the Ascidian *Polycarpa aurata* in the Coral Triangle

**DOI:** 10.1371/journal.pone.0121120

**Published:** 2015-03-23

**Authors:** J. Antonio Baeza, Carrie A. Hemphill, Raphael Ritson-Williams

**Affiliations:** 1 Department of Biological Sciences, Clemson University, Clemson, South Carolina, United States of America; 2 Smithsonian Marine Station at Fort Pierce, Smithsonian Institution, Fort Pierce, Florida, United States of America; 3 Departamento de Biología Marina, Universidad Católica del Norte, Coquimbo, Chile; 4 Hawaii Institute of Marine Biology, University of Hawaii at Manoa, Honolulu, Hawaii, United States of America; Columbia University, UNITED STATES

## Abstract

Theory predicts that monogamy is adaptive in symbiotic crustaceans inhabiting relatively small and morphologically simple hosts in tropical environments where predation risk away from hosts is high. We tested this prediction in the shrimp *Odontonia katoi*, which inhabits the atrial chamber of the ascidian *Polycarpa aurata* in the Coral Triangle. Preliminary observations in *O*. *katoi* indicated that males were smaller than females, which is suggestive of sex change (protandry) in some symbiotic organisms. Thus, we first investigated the sexual system of *O*. *katoi* to determine if this shrimp was sequentially hermaphroditic. Morphological identification and size frequency distributions indicated that the population comprised males that, on average, were smaller than females. Gonad dissections demonstrated the absence of transitional individuals. Thus, *O*. *katoi* is a gonochoric species with reverse sexual dimorphism. The population distribution of *O*. *katoi* in its ascidian host did not differ significantly from a random distribution and shrimps inhabiting the same host individual as pairs were found with a frequency similar to that expected by chance alone. This is in contrast to that reported for other socially monogamous crustaceans in which pairs of heterosexual conspecifics are found in host individuals more frequently than expected by chance alone. Thus, the available information argues against monogamy in *O*. *katoi*. Furthermore, that a high frequency of solitary females were found brooding embryos and that the sex ratio was skewed toward females suggests that males might be roaming among hosts in search of receptive females in *O*. *katoi*. Symbiotic crustaceans can be used as a model system to understand the adaptive value of sexual and mating systems in marine invertebrates.

## Introduction

The adoption of a symbiotic lifestyle (symbiosis here defined *sensu* [1] as dissimilar organisms living together) is an important environmental adaptation in marine, freshwater, and terrestrial organisms [2–4]. In marine environments, symbiotic relationships typically are comprised of small organisms (hereafter termed symbiotic guests) residing in or on biotic refuges (e.g. macro-invertebrates including: sea anemones, ascidians, corals, sponges, sea urchins, oysters) that serve as hosts [5]. Symbiotic relationships are varied and can be classified based on, among others, the costs and benefits experienced for both symbiotic guests and hosts (e.g., parasitism, mutualism, commensalism), the number of species used by one or both entities involved in the relationship (i.e., generalists versus specialists), and the degree of interdependency between the associates (i.e., facultative versus obligate symbiosis) [5–6]. In general, symbiotic partnerships are poorly understood in tropical environments given that most research has been conducted in temperate environments and because tropical environments are characterized by highly diverse, but scarce, macro-invertebrate hosts [7].

In both tropical and temperate environments, host species vary considerably in their biology and ecology, and a wide diversity of host-use patterns has been described for symbiotic guest species [3,5]. For instance, among crustaceans, a species-rich group of marine invertebrates, some symbiotic guest species dwell in or on their hosts as monogamous pairs (e.g., *Alpheus armatus*: [8,9]; *Periclimenes ornatus*: [10]; *Pontonia margarita*: [11]; *Pontonia mexicana*: [12]; *Lysmata pederseni*: [7]) or as solitary individuals (*Ascidonia flavomaculata*: [13]; *Athanas kominatoensis*: [14]; *Allopetrolisthes spinifrons*: [15]; *Pinnaxodes floridensis*: [16]; *Inachus phalangium*: [17]). Even other guest crustaceans form large aggregations of conspecifics with no evident demographic structure (*Ancylomenes pedersoni*: [18]; *Thor amboinensis*: [19]), and a few establish well-structured groups within their hosts (e.g. eusocial shrimps *Synalpheus brooksi*, *S*. *chacei*, *S*. *filidigitus*, and *S*. *regalis*: [20–22]). Overall, this diversity of host use patterns suggests that symbiotic crustaceans may be used as a model system to study the effects of various environmental conditions on the social behavior of symbiotic organisms.

**Fig 1:**
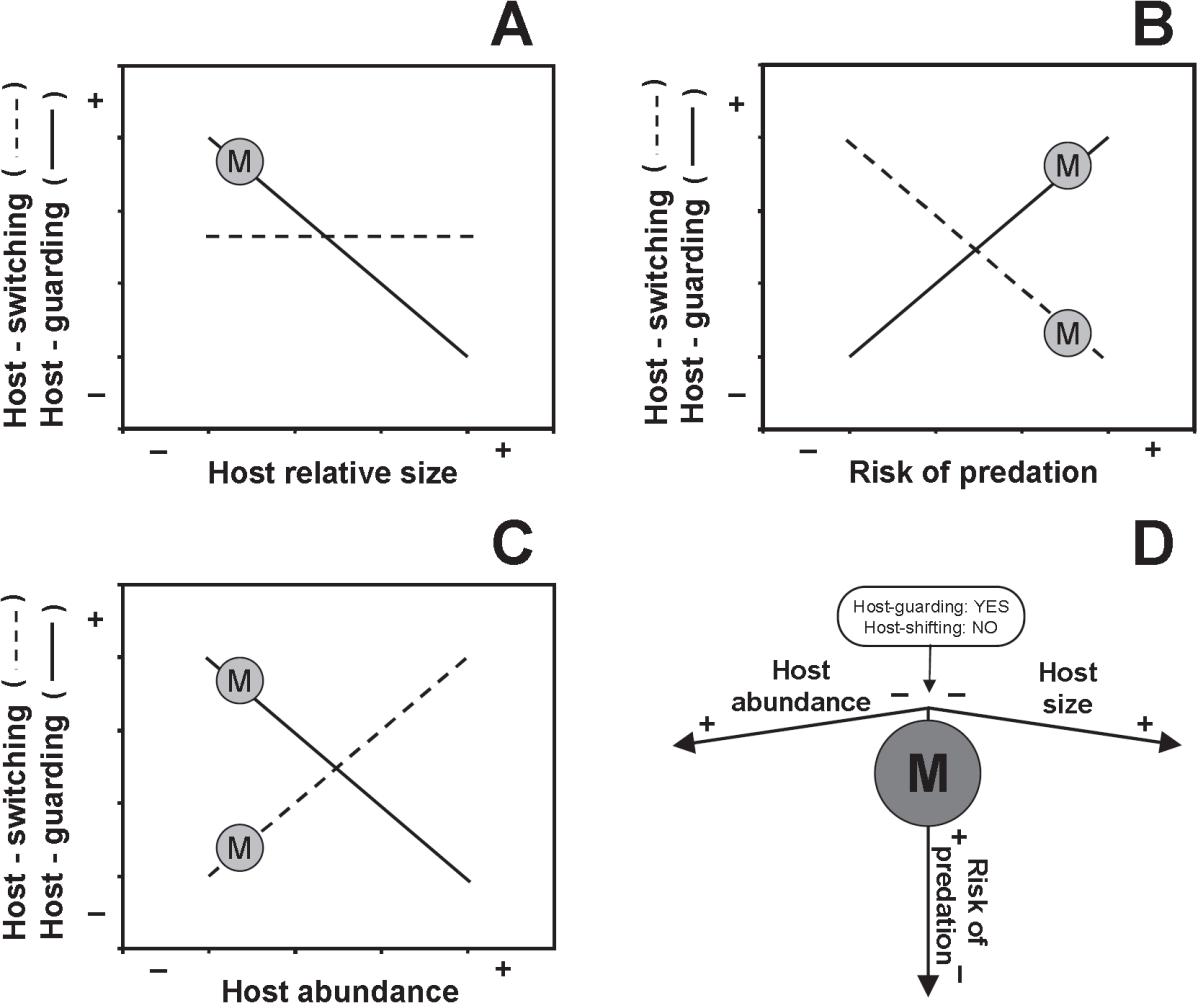
Mating systems in Symbiotic Guest Crustaceans. Monogamy, as predicted for symbiotic crustaceans, according to environmental, including host intrinsic, conditions (*sensu* model formulated in [5]). (A), (B), and (C). The different decisions on host-monopolization and host-switching (i.e., among-host individual movements) featured by symbiotic crustaceans depending upon host characteristics and predation risk away from host individuals according to an optimality (cost *versus* benefit) economical approach. For further details, see text and [5]. (A) Host-guarding (= host monopolization) is favored when host body size (relative to that of symbiotic guests) is small but not large. Host-switching is neither favored nor constrained by host body size. For further details, see text and [5]. (B) Host-guarding is favored when predation risk away from hosts is high because of the host’s value in offering protection against predators. Host switching is favored when predation risk away from hosts is low and constrained when predation risk away from hosts is high. (C) Host-guarding and host-switching are favored when host abundance is low. For further details, see text and [5]. (D) The interaction among predation pressure, host relative size, and abundance is envisioned as a tri-dimensional landscape on which different mating systems occurs. Theory predicts that monogamy is adaptive in symbiotic crustaceans inhabiting relatively small and morphologically simple hosts in (tropical) environments where predation risk away from hosts is high. Movements among hosts (i.e., host-switching) are constrained and monopolization of hosts (host-guarding) is favored in males and females due to their scarcity and because of the host’s value in offering protection against predators. Given that host space availability constraints allow only a few adult symbiotic individuals to cohabit in/on the same host, both adult males and females are expected to maximize their reproductive success by sharing 'their' dwelling (i.e., host individual) with a member of the opposite sex. Symbiotic guest individuals are typically rejected from hosts by members of the same sex, but not by members of the opposite sex. This agonistic behavior eventually leads to social monogamy and long-lasting heterosexual pairing. For details about the evolution of other mating systems see [5]. In (A), (B), and (C), monogamy = M.

What conditions explain the diversity of host use patterns and social structures exhibited by symbiotic crustaceans? Recent theoretical and empirical studies suggest that various host characteristics (e.g., host structural complexity, abundance, and body size [relative to the symbiotic guest body size] [5, 23]), predation risks away from host individuals [5], competition among symbiotic guest species using the same host species [7], and nutritional value offered by hosts to symbiotic guests [24] contribute substantially to determining the mating systems and social structures of symbiotic crustaceans. For instance, social monogamy, a mating system reported for numerous symbiotic species [5], is predicted for symbiotic guest species that inhabit scarce, small and structurally simple host species in environments in which mortality risks for symbiotic guests when away from hosts is high [5,11,12] (Fig. 1).

Under the conditions depicted above, movements among hosts are constrained and monopolization of hosts is favored in males and females due to their scarcity and because of the host’s value in offering protection against predators [5] (Fig. 1). Because space constraints allow only a few adult symbiotic individuals to cohabit in/on the same host individual, both adult males and females are expected to maximize their reproductive success by sharing “their” dwelling (i.e., host individual) with a member of the opposite sex [5,11]. Symbiotic guest individuals are typically rejected from hosts by members of the same sex, but not by members of the opposite sex. This agonistic behavior eventually leads to social monogamy and long-lasting heterosexual pairing [11]. Recent empirical studies support the notion above: numerous symbiotic crustaceans inhabiting small and discrete hosts in tropical environments where predation risk away from hosts is expected to be high are socially monogamous (e.g., *Pontonia pinnophylax*: [25]; *P*. *domestica*: [26]; *P*. *margarita*: [11,12]). A particularly interesting study also supporting the notion above is that conducted in two sibling species of shrimps from the genus *Alpheus*. *Alpheus immaculatus* features a monogamous mating system in environments where the risk of predation off hosts is relatively high (Knowlton 1980). In contrast, in its sibling species *A*. *armatus*, which inhabits environments with a lower risk of predation off hosts, males roam more frequently among host individuals in search of extra-pair copulations [27]. However, other studies have also found non-monogamous and putatively promiscuous symbiotic guest species inhabiting hosts in environments that should favor monogamy following theoretical considerations (e.g., *Ascidonia flavomaculata* [13]). Certainly, further studies are needed to fully understand the mating system and social behaviors, not only in symbiotic guest crustaceans, but in all symbiotic organisms.

The aim of this study is to test the hypothesis that symbiotic guest crustaceans inhabiting relatively small, discrete, structurally simple hosts should display a monogamous mating system [5]. As a model, we used the shrimp *Odontonia katoi* (Palaemonidae, Pontoniinae), which inhabits the branchial chamber of the ascidian *Polycarpa aurata* in the Indo-Pacific [28,29]. The ascidian host used by this symbiotic shrimp represents a small, discrete refuge that should be relatively easy to defend against intruders [5,11,23]. Also, as risk of predation is high in tropical environments where omnivorous fishes and crabs are common, it is probable that movement between ascidian hosts is costly for both males and females (see [30]). The conditions above are expected to drive a monogamous mating system in *O*. *katoi*. Here we describe the population distribution, male-female association pattern, and sexual dimorphism of *O*. *katoi* to gain better insight into the social and mating strategies of this species. If *Odontonia katoi* is socially monogamous, we predict that (1) the population distribution of *O*. *katoi* in the branchial chamber of *P*. *aurata* is non-random, (2) two individuals of *O*. *katoi* will cohabit in the same host individual with a frequency greater than that expected by chance alone, and (3) among paired shrimps, heterosexual pairs will be found more frequently than expected by chance alone. It is important to understand the sexual system of the studied species before exploring its mating system [31]. Thus, we also studied the sexual system of this species as remarkably little is known about gender expression in marine invertebrates inhabiting tropical environments [31]. Preliminary observations in *O*. *katoi* indicated that males were smaller than females, which is suggestive of sex change (protandry) in some marine invertebrates, including crustaceans [32]. Thus, we first tested for protandry in the studied species.

## Materials and Methods

### Collection of hosts and shrimps

**Fig 2:**
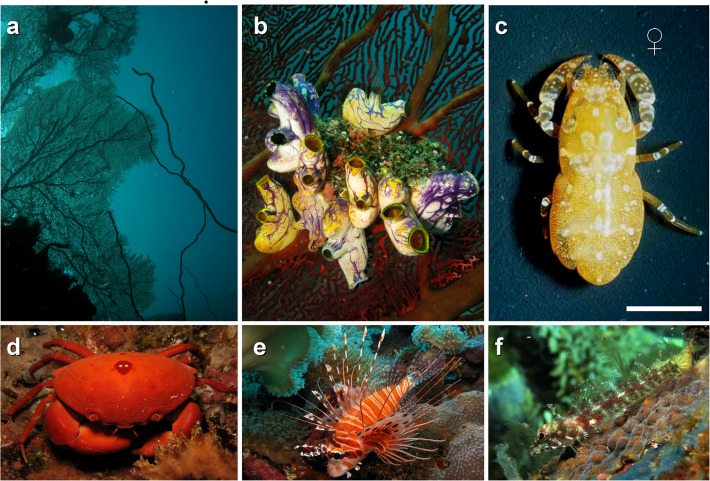
Symbiotic Guest, Hosts, and Environment. (a) Shallow subtidal at one of the study sites, Hanging Gardens Reef, Kimbe Bay, Papua New Guinea. (b) In-situ aggregation of the ascidian *Polycarpa aurata*. (c) A female of *Odontonia katoi* (photo: Charles Fransen, Naturalis, Leiden, The Netherlands). (d, e, and f) Potential predators of *O*. *katoi* at the study sites, including the xanthoid crab *Carpilius convexus* (d), lionfish *Pterois* sp. (e) and unindetified blenny (f).

Individuals of the solitary tunicate *Polycarpa aurata* were collected between June 5th and July 30th, 2009 from the subtidal (5–20 m) using SCUBA at 6 different localities: Rosemarie's Reef, Bob's Knob Reef, Hanging Gardens Reef, Luba Luba Reef, Ladi Di Reef, and Max's Reef, all located within Kimbe Bay (S 5° 19, E 150° 14´), Bismark Sea, West New Britain, Papua New Guinea. The different sampling sites were underwater rocky cliffs with a highly diverse array of sessile macroinvertebrates (Fig. 2A). The most common invertebrates observed at both sites were stony corals (*Acropora* spp.), black corals (*Stichopathes* spp., *Cirripathes* spp., and *Antipathes* spp.), various other species of ascidians, giant clams (*Tridacnia* spp.) and many unidentified sponges. *Polycarpa aurata* individuals were found either solitarily, or occasionally, in small groups (2–10 individuals) cemented directly to the rocky substrate or at the base of the sea whips or on the branches of the black corals (Fig. 2B). Species of omnivorous / predatory crabs and fishes (known to prey upon crustaceans: [30]) observed at all study localities included various species of xanthoid crabs as well as search-and-catch (e.g., wrasses *Halichoeres* spp.) and sit-and-wait fish predators (the lionfish *Pterois* spp., the scorpionfish *Scorpaena* spp., unidentified gobies, blennids and damselfish) (Fig. 2D-F).

Haphazardly collected ascidians along a 30 m transect located at 5–20 m depth at each study site were individually placed in Ziploc bags and transported to the research vessels Kanai 1 or Kanai 2 of the Mahonia Na Dari Research Station, Kimbe, Papua New Guinea. In the research vessels or at the research station, ascidians were gently dissected with a scalpel, and each shrimp found in the pharyngeal basket was fixed in 95% Ethanol for transportation to the laboratory. In the laboratory, the maximum length and width of each tunicate were measured with a caliper to the nearest 0.1 mm. The number of shrimps per host was also recorded (see below).

The study locations are traditionally owned and protected by local native villagers. Access and permission for sampling at the different localities above were generously granted by the Ritu—Bitokara authorities. Our field studies did not involve endangered or protected species.

### The sexual system of *Odontonia katoi*

To examine the sexual system of *O*. *katoi*, observations on reproductive anatomy were made on a total of 99 specimens previously extracted from ascidians. First, the carapace length (CL), the length of the largest of the first pair of chelipeds, and the second abdominal segment (maximum lateral length of the right pleuron) of all shrimp were measured under the stereomicroscope to the nearest 0.01 mm. Next, the sex of each shrimp was determined based on appendices masculinae on the base of the endopod of the second pleopods (present in males but absent in females) [11,33,34]. Then, each female shrimp was classified according to the presence or absence (ovigerous or non-ovigerous) of embryos beneath the abdomen. Finally, the gonads of 20 randomly selected shrimp classified as males or females (see results) based on the external characters mentioned above were dissected to confirm sex of each shrimp.

During measurements and dissections, we focused on recognizing 'transitional' individuals in our sample: shrimp with a combination of male and female traits (e.g. shrimp with appendices masculinae on the second pleopods [male character] and maturing ovaries or brooding eggs [female characters]). These 'transitional' individuals have been reported before for various other shrimp species that undergo shifts in sex allocation during their lifetime and the presence of these 'transitionals' represents a reliable indication of sex change [7,11,19,33,35–37].

### Sexual dimorphism in *Odontonia katoi*

In caridean shrimps from the family Palaemonidae, including representatives from the genus *Odontonia*, the second pair of thoracic appendages bears the largest of the two pairs of claws [38]. These structures serve as weapons during intra-sexual interactions or for inter-sexual communication [39]. In turn, the pleura of the second abdominal segment are greatly enlarged and help protect the embryos (i.e., from physical abrasion) carried by females beneath their abdomen [38].

We examined whether the chelipeds in the second pair of pereopods and the pleuron of the second abdominal segment increase linearly with body size in males and females of *O*. *katoi* following [11]. In short, the relationship between the length of the merus of the largest second cheliped or the length of the pleuron of the second abdominal segment and body size of shrimps (CL, mm) was examined using the allometric model y = ax^b^ [40,41]. The slope b of the log-log least-squares linear regression represents the rate of exponential increase (b>1) or decrease (b<1) of the cheliped and abdominal segment with a unit of increase in body size of shrimp. A t-test was used to determine if the estimated slope b deviates from the expected slope of unity [40–42]. If the cheliped or the abdominal pleuron grow more or less than proportionally with a unit increase in body size of shrimp, then the slope should be greater or smaller than the unity, respectively [40,41].

### Host use pattern of *Odontonia katoi*

To test for social monogamy in *O*. *katoi*, we examined the host use pattern of this shrimp, which includes a description of its population distribution, male–female association pattern, and host–shrimp body size relationships. First, we examined whether or not symbiotic shrimp live solitarily, form aggregations, or pairs inside the host species. For this purpose, we examined whether or not the distribution of *O*. *katoi* in the ascidian host (i.e., the frequency of occurrence of hosts without shrimp and with different numbers of shrimp) differed from a random distribution. We compared the observed distribution (i.e., frequency of occurrence of hosts with zero, one, two, three or more shrimp) with the Poisson random distribution [43]. Significant differences between the distributions were examined using a Chi–square test of goodness–of–fit [44]. When significant differences were observed, specific frequencies between the observed and expected distributions were compared by subdivision of the Chi–square test and using the sequential Bonferroni correction to control for false discovery rate [45].

A small percentage of tunicate hosts were found to contain pairs of shrimp (see results). To determine whether the sexes are randomly distributed among pairs inhabiting the same host, the observed distribution was compared with the binomial distribution. The expected random frequencies of distribution of the different sexes were calculated based on the proportion of males and females recorded in the population. A Chi-square test of goodness of fit was used to inspect for significant differences between the distributions as indicated above [44].

## Results

### The sexual system of *Odontonia katoi*

A total of 41 out of 99 shrimp examined were classified as males, considering only their external morphology. All of these male shrimp had appendices masculinae and internae on the endopod of the second pair of pleopods (S1 Fig.). Appendices masculinae invariably had several slender setae at its ventral and terminal portion (S1A, S1B Fig.). Dissections confirmed the presence of paired testes with lateral vas deferens in nine out of a random sub-sample of 10 shrimp classified as males based on external characters. One of the dissected males in this random sub-sample harbored a parasitic isopod (probably, family Entoniscidae) instead of testes on top of the hepatopancreas.

A total of 51 individuals out of 99 shrimp examined were classified as females considering their external morphology. All these shrimp lacked appendices masculinae on the endopod of the second pair of pleopods (S1C, S1D Fig.). Of these 51 females, 25 (49.02%) carried embryos in different stages of development. Dissections invariably demonstrated the existence of paired ovaries in a sub-sample of 10 shrimp classified as females (7 and 3 individuals with and without embryos, respectively). Ovaries were located above the hepatopancreas and projected to posterior into the first abdominal segment.

**Fig 3:**
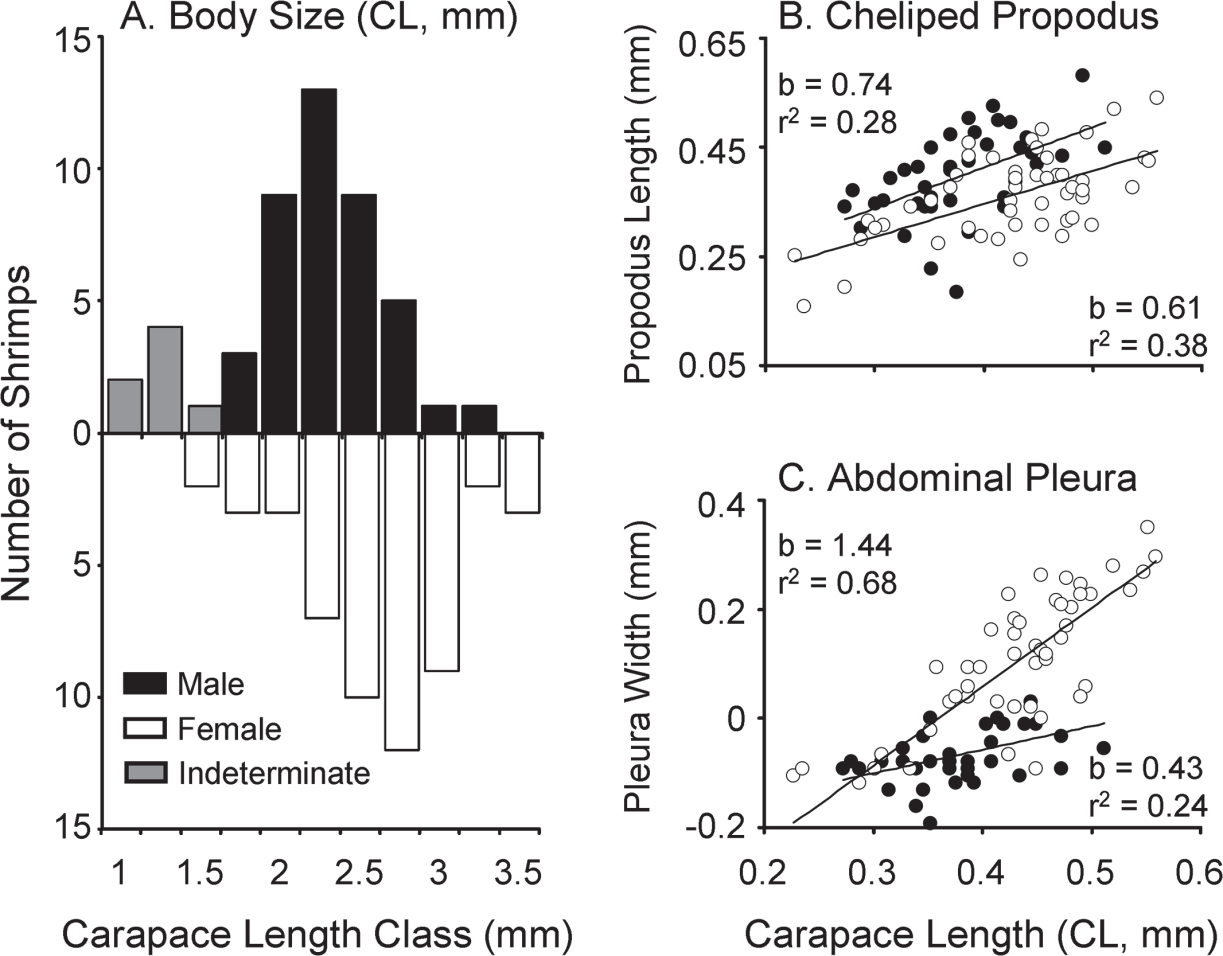
Sexual dimorphism in *Odontonia katoi*. (A) Size frequency distribution of body size (CL) in males and females. Measurements are in mm. (B) Relative growth of major cheliped propodus length as a function of carapace length in males and females of *O*. *katoi*. (C) Relative growth of pleuron of second abdominal segment as a function of carapace length in males and females of *O*. *katoi*. Lineal regression equation obtained previous log-log transformation of the data are shown for each sex on Table 1.

A total of 7 individuals out of 99 shrimp examined were not sexed due to their small body size (1.22 < CL < 1.69 mm; Fig. 3A). During dissections, we did not observe any 'transitional' shrimp; individuals with a combination of male and female external and/or internal characters.

**Table 1:** Relative growth of selected structures in males and females of *Odontonia katoi*.

Sex	*y*	*x*	Regression	*r* ^*2*^	SE_s_	*t*_s_	*P*	Allometry	Sexual Dimorphism
Males	PL	CL	y = 0.7447 x + 0.1149	0.278	0.1972	1.29	> 0.05	0	M > F
Females	PL	CL	y = 0.6066 x + 0.1036	0.381	0.1117	3.52	0.01	-	
Males	AL	CL	y = 0.4310 x − 0.2297	0.236	0.1241	4.59	0.01	-	M < F
Females	AL	CL	y = 1.4431 x − 0.5193	0.679	0.1416	3.13	0.01	+	

The regression equations, correlation coefficients (*r* ^*2*^, adjusted for d.f.), standard errors of the slopes (SE_s_), and the allometric status of each studied variable are shown (CL, PL, and AL = carapace length, length of the merus of the major cheliped, and length of the second abdominal pleuron, respectively). ANCOVAs were used to test for differences in PL and AL between males and females. See text for details.

### Sexual dimorphism in *Odontonia katoi*

The CL of male and female shrimp varied between 1.88 and 3.25 mm (mean ± SD, 2.43±0.34) and between 1.69 and 3.63 mm (2.70±0.46), respectively. The carapace length (CL) of females was larger than that of males (Kruskal-Wallis test [variances were heterogeneous]; Z = -3.267, *P* = 0.001] indicating reverse sexual dimorphism (males < females) with respect to body size in *O*. *katoi* (Fig. 3A).

A positive correlation was detected between CL and the length of the propodus of the major cheliped in shrimp of both sexes, as well as between CL and the width of the right pleuron of the second abdominal segment in both sexes (Fig. 3B,C). In males, the growth of the major cheliped was isometric with respect to body size; the slope of the relationship between male CL and major cheliped length did not differ significantly from unity (b = 0.75, *P* > 0.05). In females, the propodus of the major cheliped presented negative allometry; the slope of the relationship between female CL and propodus length was significantly smaller than unity (b = 0.61, *P* = 0.01). An analysis of covariance (ANCOVA) indicated a significant effect of sex (*F* = 20.05, d.f. = 1, 88, *P* < 0.0001) and CL in propodus length (*F* = 37.74, d.f. = 1, 88, *P* <0.0001), and the interaction term of this analysis was not significant (*F* = 0.3941, d.f. = 1, 88, *P* = 0.5319). Therefore, at any given body size, the size of the major cheliped (expressed as propodus length) is slightly larger in males than in females of *O*. *katoi* (Fig. 3B) (Table 1).

In males, the pleuron of the second abdominal segment presented negative allometry; the slope was significantly smaller than unity (b = 0.43, *P* = 0.01), whereas in females, the same structure presented positive allometry (b = 1.44, *P* = 0.01). An ANCOVA indicated a significant effect of sex in pleuron length (*F* = 67.45, d.f. = 1, 91, *P* < 0.0001). The ANCOVA also detected an effect of CL in pleuron length (*F* = 74.75, d.f. = 1, 91, *P* < 0.0001), and the interaction term was significant (*F* = 21.80, d.f. = 1, 91, *P* < 0.0001). Thus, the absolute size of the second abdominal pleuron and the growth rate of this structure were greater in females than in males of *O*. *katoi* (Fig. 3C) (Table 1).

### Host use pattern of *Odontonia katoi*

Between 19 and 21 specimens of the ascidian *Polycarpa aurata* were collected from the different sampling sites in Kimbe Bay, Papua New Guinea (Table 2). The average (± S.D) length of the sampled ascidians varied between 48.58 (± 9.15) mm and 54.61 (± 13.55) mm at Maxx Huva and Bob’s Knob, respectively (Table 2). The population size distribution of the ascidians was similar among the different sampling sites and there was no significant difference in host sizes among localities (ANOVA; F = 0.89, df = 5,119, *P* = 0.4927).

**Table 2:** Characteristics of the association between the shrimp *Odontonia katoi* and its ascidian host *Polycarpa aurata*.

Study Site				HW (mm)			Shrimp Density			Shrimp CL (mm)		
	NH	P	NS	Avg	SD	Range	Avg	SD	Range	Avg	SD	Range
Rosemarie's	19	52.6	17	33.78	6.03	20.9–44.3	0.895	0.937	0–2	2.41	0.54	1.69–3.53
Bob's Knob	20	55.0	16	36.38	9.02	19.0–50.2	0.800	0.834	0–2	2.59	0.58	1.34–3.13
Hanging Gardens	21	47.6	11	33.76	5.66	25.0–47.0	0.524	0.602	0–2	2.20	0.49	1.22–2.88
Maxx Huva	19	42.1	12	32.74	5.36	25.0–44.0	0.632	0.831	0–2	2.65	0.45	2.13–3.31
Luba Luba	21	52.4	14	36.10	8.63	18.0–49.0	0.667	0.730	0–2	2.59	0.54	1.69–3.56
Lady Di	20	60.0	14	36.50	8.02	27.0–49.0	0.700	0.657	0–2	2.51	0.32	1.97–2.97

NH = number of ascidians collected at each locality, P = shrimp prevalence (%), NS = total number of shrimps collected at each locality, HW = average (Avg) +/- standard deviation (SD) tunic width of ascidians (mm) collected at each locality, Shrimp Density = average (Avg) +/- standard deviation (SD) of the number of shrimps collected per ascidian host at each locality, Shrimp CL = average (Avg) +/- standard deviation (SD) of carapace length of shrimps collected per ascidian host at each locality.

**Fig 4:**
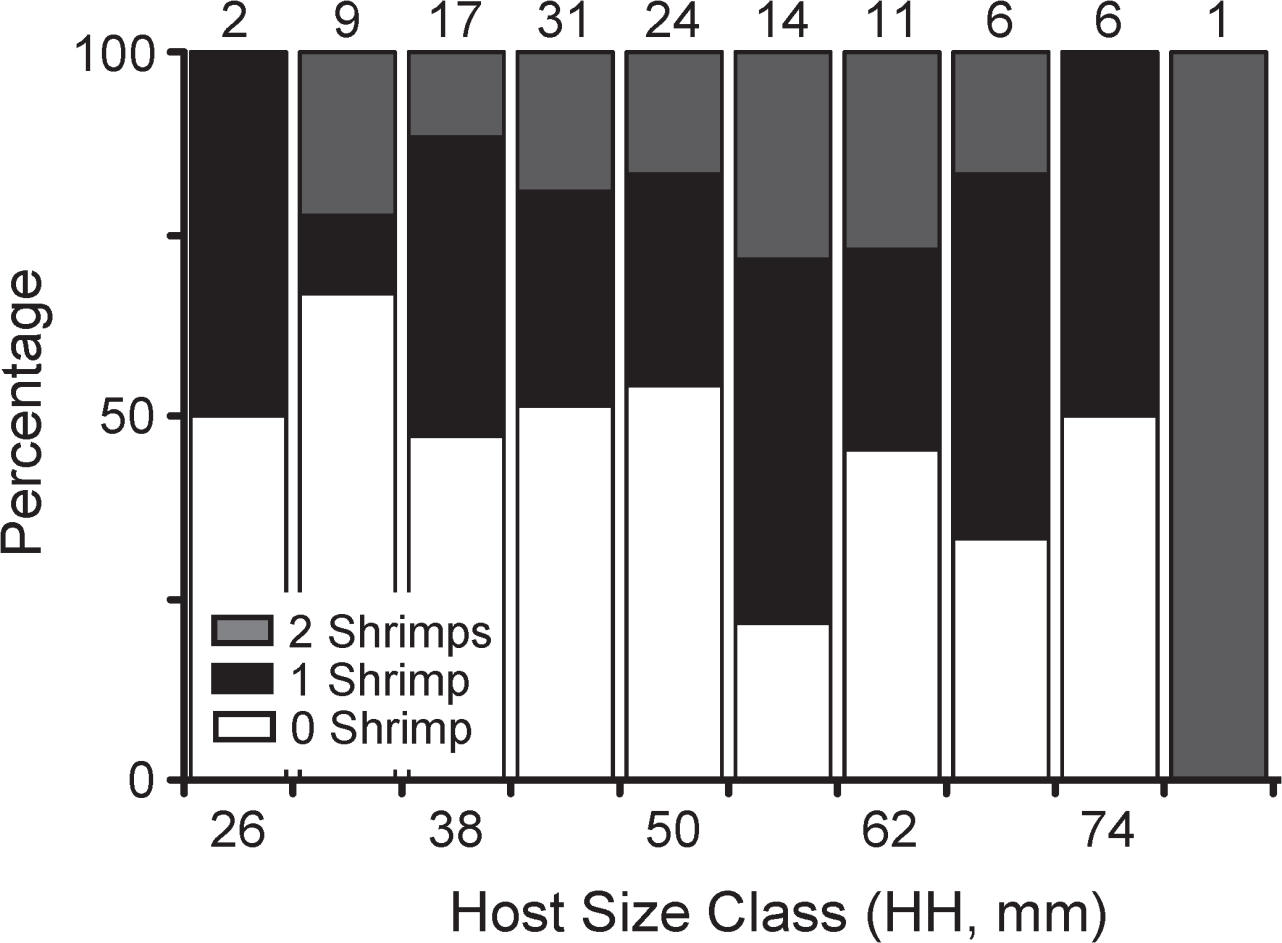
The effect of host body size in host use by *Odontonia katoi*. Frequency of occurrence of *Odontonia katoi* shrimps in ascidian host individuals of different size classes at Kimbe Bay, Papua New Guinea (see Results for further details).

Frequency of occurrence of *Odontonia katoi* in *Polycarpa aurata* varied between 42.1% and 60.0% in Maxx Huva and Lady Di, respectively (Table 2). The difference in the frequency of occurrence of shrimp in ascidians among the different study localities was not significant (χ^2^ test of independence; χ^2^ = 8.56, df = 5, *P* = 0.1278). Ascidians from all body sizes harbored shrimp with similar frequencies (contigency table analysis; χ^2^ = 5.73, df = 8, *P* = 0.66) (Fig. 4).

**Fig 5:**
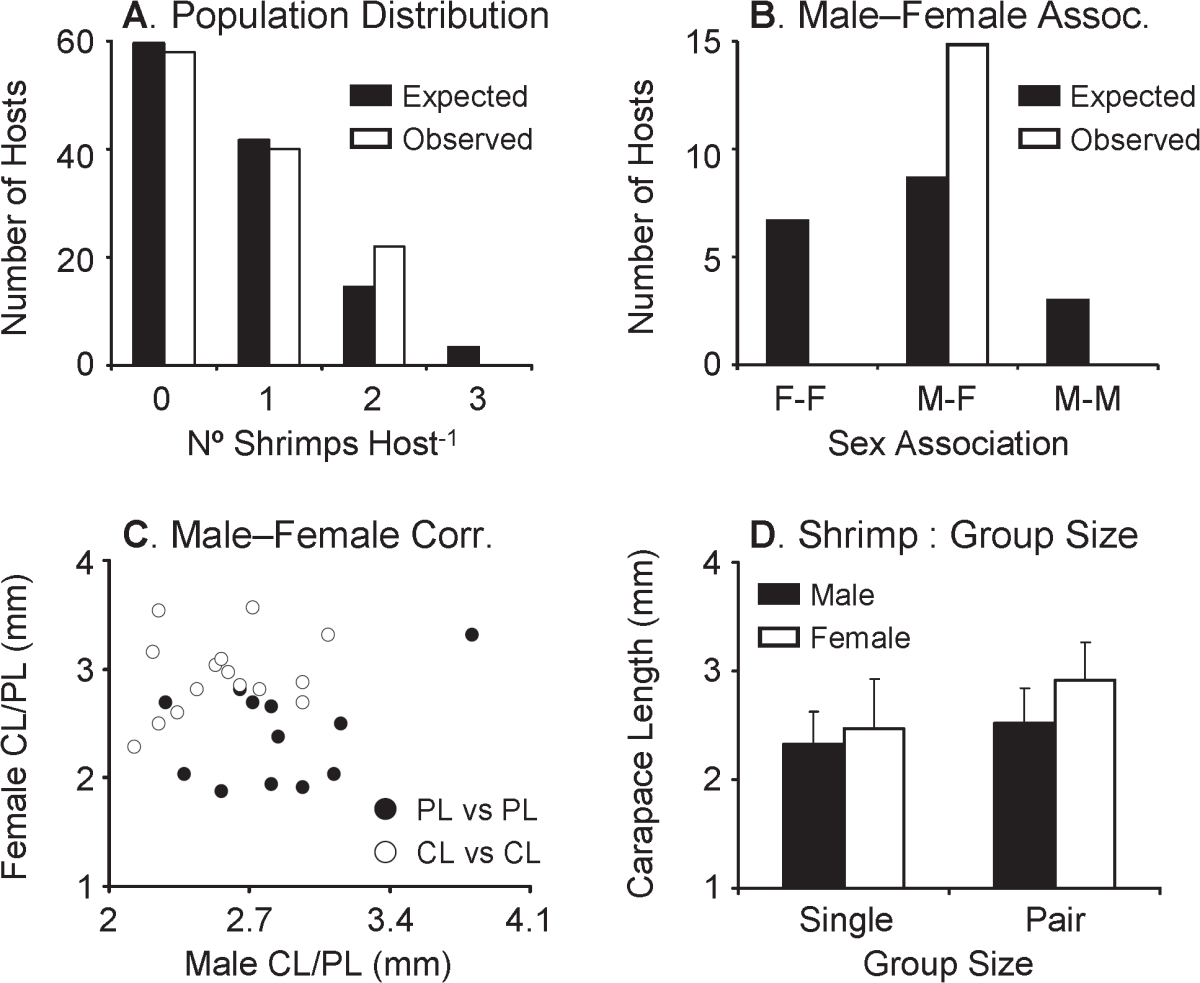
Host use pattern of *Odontonia katoi* (combined data from different sampling locations) at Kimbe Bay, Papua New Guinea. (A) Population distribution of *O*. *katoi*. Observed frequency of shrimps on hosts did not differ significantly from an expected Poisson random distribution. (B) Male-female association pattern of *O*. *katoi* found as pairs inside the atrial chamber of *Polycarpa aurata*. Observed frequency of heterosexual pairs differs significantly from the expected binomial random distribution. (C) Relationship between carapace length (CL) and cheliped length (PL) of males and females of *O*. *katoi* found as heterosexual pairs inside the atrial chamber of *P*. *aurata*. (D) Carapace length (mean and standard deviation [bars]) of male and female shrimps occurring either solitarily or in pairs inside the atrial chamber of *Polycarpa aurata*. Males and females were smaller when found solitarily compared to when found in pairs. See results for further details.

For the analysis of population distribution and male-female association pattern in *Odontonia katoi*, the data were pooled together because of the absence of differences among sampling sites in the body size of hosts and the occupancy of hosts by shrimp. A total of 28 males, 46 females and 2 undetermined shrimp were retrieved from ascidians in all study sites. The sex ratio (= males / males + females) was biased towards females in the population (sex ratio = 0.38; Fisher's Exact test, P < 0.001). The number of shrimp per host varied between 0 and 2 with a mean of 0.70 ± 0.76. The population distribution of *Odontonia katoi* in ascidians displayed a random pattern (Chi-square test of goodness of fit, χ^2^_2_ = 1.05, *P* = 0.5916). In general, host individuals harbored either only one or two shrimp with frequencies similar to that expected by chance alone and never three or more shrimp (Fig. 5A).

A total of 40 ascidians harbored a single shrimp; 11 males and 29 females (11 of which were brooding embryos). A total of 19 ascidians harbored two shrimp. Pairs of shrimp inhabiting hosts consisted of one mature male and one mature female or one mature male or mature female with one immature shrimp of indeterminate sex (mature male—mature female pairs = 15, mature female and one juvenile = 2, mature male and one juvenile = 2). No ascidian harbored two mature male shrimp or two mature female shrimps (Fig. 5B). Taking into consideration the binomial distribution and the relative abundance of males and females in the studied populations, the number of hosts harboring heterosexual pairs expected by chance alone would have been 8.93 out of 15 hosts. Therefore, paired shrimp were found as heterosexuals more frequently than expected by chance alone.

There was no correlation between the body size (CL) of males and females found as pairs (t-test; t = 0.96, df = 1,14, *P* = 0.3538) (Fig. 5C); only 6.64% of the variation in male CL was explained by female CL (r^2^ = 0.0644). In 13 out of the 15 heterosexual pairs, the female was larger than the male, and in two out of 15 of the heterosexual pairs, the male was larger than the female. On average, females were 18.87% (± 15.83) larger than their male companions. Also, there was no correlation between the size of the major cheliped of males and females found as pairs (t-test; t_1,14_ = 1.22, *P* = 0.2455); only 10.22% of the variation in female cheliped size was explained by male cheliped size (r^2^ = 0.1022) (Fig. 5C). Of the females found with a male in the same host, 14 out of 15 (93.33%) were brooding embryos.

Solitary and paired males were, respectively, 2.33 ± 0.30 mm CL (range: 1.94 to 2.81 mm CL) and 2.52 ± 0.32 mm CL (range 1.88–3.09 mm CL). Solitary and paired females were, respectively, 2.47 ± 0.46 mm CL (range: 1.69–3.09 mm CL) and 2.92 ± 0.35 mm CL (range 2.28 to 3.56 mm CL). A two-way ANOVA indicated a significant effect of group size (solitary < paired) in shrimp body size (*F* = 12.096, d.f. = 1, 69, *P* = 0.0009). As expected, the ANOVA also detected an effect of sex (females > males) in shrimp body size (*F* = 8.2408, d.f. = 1,69, *P* = 0.0055), and the interaction term was not significant (*F* = 1.87, d.f. = 1, 69, *P* = 0.1766) (Fig. 5D).

There was a statistically significant correlation between host size and shrimp size, regardless of the presence or absence of other shrimp in the same host, for males, but no statistically significant correlation for females (t-test; t = 2.14, df = 1,28, *P* = 0.0412, and t = 1.24, df = 1, 40, *P* = 0.2239, for males and females, respectively). Though the correlation for males was statistically significant, only 14.5% of the variation in male body size was explained by host size (r^2^ = 0.1455).

## Discussion

### The sexual system of *Odontonia katoi*

In *O*. *katoi*, the different studied populations comprised males that were, on average, smaller than females. At a first glance, this difference in body size between the sexes agrees with expectations for protandric hermaphrodites [32,38]. However, during dissections, we never observed transitional individuals having characteristics of both males and females (e.g., appendices masculinae and ovaries). The above rules out simultaneous hermaphroditism as the sexual system of the studied shrimp and also demonstrates the absence of individuals in the process of changing sex ('transitionals') in natural populations. Transitional individuals have been reported before for various other shrimp species that undergo shifts in sex allocation during their lifetime and do represent a reliable indication of sex change in marine invertebrates, including shrimps [11,19,33,35,36,46]. In our samples, individuals that were categorized either as males or females based on external features had, almost invariably, male or female gonads, respectively. These observations show that the studied shrimp is a gonochoric species; individuals of *O*. *katoi* are born, reproduce and remain as either males or females during the rest of their life.

Admittedly, it could be argued that *O*. *katoi* does change sex, but that the transition from small males to larger females in this species is facultative and not obligatory and that it occurs rarely, as reported before for other caridean shrimps. For instance, in the sex changing shrimp *Crangon franciscorum*, laboratory experiments have demonstrated morphological evidence of sex change only in 2 out of 40 experimental males [36]. Similarly, laboratory observations over a period of time extending for ∼ 8 months have demonstrated that less than 2% of the males in a population of the protandric shrimp *Crangon crangon* turn to females [47]. If sex change in *O*. *katoi* is facultative rather than obligatory and the probability of males changing sex is low (e.g., as low as in *C*. *crangon* and *C*. *franciscorum*), then our low sample size (a total of 99 shrimp) would allow sex change in this species to go undetected. Future studies could track individual shrimp to test the possibility of facultative sex change in *O*. *katoi*. Nonetheless, all presently available information suggests that *O*. *katoi* is gonochoric.

Importantly, the Infraorder Caridea, to which *O*. *katoi* pertains, is the most diverse in terms of sexual systems among decapod crustaceans [38]. In addition to gonochorism (e.g., this study) and strict protandry (e.g., *Merguia rizhophorae*: [46], *Thor amboinensis*: [19]), other species are reported to display mixed protandry; populations in which individuals that undergo sex change coexist with other conspecifics that mature either as pure males or females and that do not change sex (e.g., *Pandalus* spp.: [48]; *Crangon crangon*: [47] but see [49]; *Thor manningi*: [33]). Lastly, protandric simultaneous hermaphroditism has been demonstrated in members from the genera *Lysmata*, *Exhippolysmata* and *Parhippolyte* [37,50,51]. In the above species, juveniles invariably mature as functional male individuals first and later become functional simultaneous hermaphrodites [50–52]. Further detailed studies on the lifestyle and sexual system of more species of caridean shrimps and the development of a comprehensive molecular phylogeny in this clade of shrimps [53–55] are necessary to elucidate the fascinating evolutionary history of gender expression in the Caridea.

### Is *Odontonia katoi* socially monogamous?

We hypothesized that *O*. *katoi* was socially monogamous, and thus, we expected that (1) the population distribution of *O*. *katoi* in the branchial chamber of *P*. *aurata* was non-random, (2) a pair of *O*. *katoi* will cohabit the same host individual with a frequency greater than that expected by chance alone, and (3) among paired shrimps, heterosexual pairs will be found more frequently than expected by chance alone. Our results mostly are in disagreement with our theoretical considerations. Although most shrimps inhabiting ascidians as pairs comprised heterosexual couples (in agreement with prediction [3] above), the overall population distribution of *O*. *katoi* in its ascidian hosts did not differ significantly from a random (Poisson) distribution, and shrimp inhabiting the same host individual as pairs were found with a frequency similar to that expected by chance alone. This is in contrast to that reported for other socially monogamous crustaceans in which, (heterosexual) pairs are found in host individuals more frequently than expected by chance alone (e.g., *Pontonia margarita*: [11]; *Pontonia sp*.: [56]; *Pontonia mexicana*: [12]). Below, we discuss additional characteristics of the association between *O*. *katoi* and *P*. *aurata* indicating that the studied symbiotic shrimp is not monogamous but rather promiscuous.

In *O*. *katoi*, the weak (in males) or lack (in females) of a relationship between shrimp and host body size as well as between the body sizes of the relatively few paired males and females in the population further argue against the notion of monogamy. Often, in symbiotic and monogamous crustaceans, in which male and female symbiotic guests develop a long lasting association with one another as well as with their host individuals, a tight correlation between host and symbiotic guest body size is found [11,57,58]. Growth limitations imposed by host individuals over their long-term resident symbiotic guests is typically invoked to explain such tight host-shrimp body size relationship (see [11] and references therein). Our results in *O*. *katoi* contrast to that reported for symbiotic monogamous species; if *O*. *katoi* was a socially monogamous species then, a tight relationship between shrimp and host body size would have been found within the few heterosexual pairs found, similar to that reported for other monogamous species in which symbiotic guests stay for long periods of time in/on their host individuals [11,37,57]. The weak correlation between the body size of shrimp and the host individuals harboring them additionally suggests that male (and/or female) shrimps might be shifting among host individuals frequently as reported for other promiscuous symbiotic crustaceans (e.g., *Liopetrolisthes mitra*: [59,60]).

Also interpreted as a consequence of growth limitations on symbiotic guests imposed by hosts is the widely reported tight correlation between paired male and female individuals in symbiotic monogamous crustaceans [11,57,58,61]. In the symbiotic and monogamous *Pinnixa transversalis* and *Pontonia margarita*, male body size explains 77.6% and 63.8%, of variation in female body size, respectively [11,57]. In *O*. *katoi*, no correlation between male and female body size was found among the few shrimp inhabiting hosts as heterosexual pairs. This absence of size-assortative pairing in *O*. *katoi* suggests that the association between males and females is not temporally stable and that males (and probably females) do not cohabitate in one host individual for long periods of time. Instead, male and/or female shrimp might be shifting among host individuals and changing sexual partners regularly as reported for other non-monogamous species in which no correlation between paired male and female body size has been reported [59,60].

Lastly, the observed pattern of sexual dimorphism in *O*. *katoi* also suggests that this shrimp does not exhibit a socially monogamous mating system. On one hand, in agreement to that reported for other socially monogamous shrimps (e.g., *Pontonia margarita*: [11]; *Pontonia sp*.: [56]), males of *O*. *katoi* were, on average, smaller than females, and the major cheliped of males did not exhibit positive allometry. Small size of males in monogamous species is expected due to the low intensity of intrasexual competition in this mating system [5,62]. However, although the major cheliped did not exhibit positive allometry, males of *O*. *katoi* had a major cheliped larger than that of females at any given body size. Such sex-specific difference in resource allocation to chelipeds disagrees with prediction of low sexual dimorphism in terms of weaponry in monogamous species [5]. Large claws in males compared to females are uncommon in monogamous crustaceans, but common in non-monogamous shrimps where males frequently compete for females through agonistic interactions [5,38]. The larger chelipeds in males of *O*. *katoi* (compared to females) suggest that males are competing for receptive females via overt aggression. For instance, if some males switch among hosts (see above and below), when resident and intruder males meet, cheliped size likely determines the winner of the agonistic interaction and access to the receptive females [63,64].

### Is *Odontonia katoi* a promiscuous species?

Altogether, the above information strongly argues against the notion of social monogamy in *O*. *katoi*. Rather, our results suggest that *O*. *katoi* exhibits a promiscuous mating system with males, and possibly females, moving among host individuals in search of receptive sexual partners (see [5]). Indeed, an additional line of evidence suggesting that *O*. *katoi* is promiscuous and that males might be roaming around among host individuals in search of sexual partners is the observation of solitary females brooding eggs. In Caridean shrimps, including *O*. *katoi*, females do not store sperm and need to be inseminated short after molting to fertilize a new batch of eggs [38]. Thus, it is difficult to explain the existence of solitary females brooding eggs in this shrimp if males were not roaming among hosts in search of sexual partners. The existence of solitary females brooding embryos in *O*. *katoi* agrees with that reported for other symbiotic species in which males move among hosts in search of receptive females (e.g., the sea urchin dwelling shrimp *Liopetrolisthes mitra*: [59]). Our results also contrast to that reported for other socially monogamous symbiotic species in which females do not brood embryos when found solitarily in/on their hosts (i.e., the caridean shrimp *Paranchistus pycnodontae* symbiotic with the winged pearl oyster in the Indo-Pacific [31]).

Lastly, the female-skewed sex ratio in the studied solitary population of *O*. *katoi* represents another line of reasoning indicating that this symbiotic shrimp is not monogamous and suggests that males, but not necessarily females, might be leaving hosts (at least temporarily) in search of mating partners. Males and females are found in similar proportions in populations of symbiotic crustaceans that exhibit a monogamous mating system [11,57]. In *O*. *katoi*, sex specific differences in mortality rates by predators, driven by a greater propensity of males (compared to females) to switch among host individuals, could lead to the observed sex ratio being skewed towards females in the population. Female-skewed sex ratios have been reported before in other symbiotic and free-living crustaceans in which males frequently roam among hosts in search of receptive females [13,14].

Unfortunately, this study does not allow further conclusions about other aspects of the reproductive behavior of this species. For instance, we do not know if males abandon females immediately after insemination, or if females are guarded by males after copulation. The fact that many paired males shared their host with brooding females suggests that males might be guarding females for some unknown period of time before and after insemination, as suggested for another symbiotic shrimp, *Athanas kominatoensis*, in which males roam among hosts in search of receptive females [14]. Post-copulatory mate guarding behavior has been demonstrated for both solitary crustaceans (e.g., the solitary crab *Inachus*. *phalangium*: [65]) and monogamous crustaceans (e.g., the socially monogamous shrimp *Hymenocera picta*: [66]). While the evidence presented above suggests that *O*. *katoi* is a promiscuous species with males and possibly females frequently roaming between ascidians in search of receptive sexual partners, further observations need to be conducted to gain a better understanding of this mating system. Field observations and experiments that might help in revealing the mating strategy of *O*. *katoi* are not feasible at this time due to the remote habitat of these organisms. However, laboratory observations on the movement patterns of shrimp might help in revealing more details about the reproductive behavior of both males and females of this species.

### Conclusions

*Odontonia katoi* is a gonochoric species with reverse sexual dimorphism. Importantly, cases of reverse sexual dimorphism have been used before as 'evidence' of sex change (protandry) in some marine invertebrates, including symbiotic crustaceans [32]. Thus, one implication from this study is that reverse sexual dimorphism cannot be used as proxy for sex change in marine invertebrates. The sexual systems of symbiotic crustaceans have rarely been studied in detail (for exceptions, see [7,11,14,52], among a few others). Most commonly, symbiotic crustaceans are assumed to be gonochoric, although recent studies have demonstrated partial sex change [14], strict protandry [19] and protandric simultaneous hermaphroditism [37] in various symbiotic shrimps. The sexual system of symbiotic crustaceans deserves more attention.

Our results strongly argue against social monogamy in *O*. *katoi*. At first glance, the absence of pairing appears to be suboptimal and maybe maladaptive in the studied species given environmental conditions; *Odontonia katoi* inhabits a relatively small, and morphologically simple ascidian host in tropical environments where predation risk away from hosts is high [5]. Thus, we expected *O*. *katoi* to be socially monogamous. On the other hand, the number of hosts not harboring shrimps was relatively high (40–58%) and those ascidians not hosting shrimps were both among the smallest and largest ascidians collected during this study. The above suggests that hosts might not necessarily represent a limiting resource (i.e., scarce refuge) to shrimps; the ascidian host population can putatively support a larger number of symbiotic guest shrimps and all host individuals represent a suitable shelter for *O*. *katoi*. Indeed, the relatively low occupancy of ascidians by shrimps, the apparent lack of host size constraints for shrimp utilization, and the apparent gregarious distribution of ascidian hosts (see methods) might explain the putatively promiscuous mating system herein suggested for *O*. *katoi*: host availability and gregarious behavior might favor pure search mating strategies in symbiotic guest species even in environments with high predation risk like shallow and diverse coral reefs in the Coral Triangle. Further experimentation could help revealing the relative importance of host distribution pattern (e.g., uniform vs. gregarious) and occupancy rates in determining optimal mating strategies in symbiotic crustaceans.

The mating system of no other species in the same genus *Odontonia* has been studied. However, a few other species in closely related genera whose host-use patterns have been described are socially monogamous (*Pontonia margarita*: [11], *Pontonia domestica*: [26]). Shrimps from the genus *Odontonia* and closely related genera (e.g., *Ascidonia*, *Pontonia*, *Rostronia*) inhabit hosts with differing biology and ecology [28,29]. Because of the differing ecology of the host species, shrimps from the closely related genera above should also display different mating systems. Symbiotic shrimps in the subfamily Pontoniinae are proposed as a model group to understand the evolution of mating systems in species inhabiting discontinuous refuges.

## Supporting Information

S1 Fig Second pleopods of *Odontonia katoi*. (A) Endopod (en) and exopod (ex) of second pleopod in male individual. The inner margin of the second pleopod in males exhibit an appendix masculina (am) bearing spines and appendix interna (ai). (B) Close-up of the appendix masculina (am) bearing spines and appendix interna (ai) bearing coupling hooks (cc) in males. (C) Endopod (en) and exopod (ex) of second pleopod in female individual. The inner margin of the second pleopod in females exhibit an appendix interna (ai) but lacks an appendix masculina (am). (D) Close-up of the appendix appendix interna (ai) bearing coupling hooks (cc) in females.
(TIF)
