# Supplementary figures and images for: The Sexual and Mating System of the Shrimp Odontonia katoi (Palaemonidae, Pontoniinae), a Symbiotic Guest of the Ascidian Polycarpa aurata in the Coral Triangle
Source: PLoS One. 2015 Mar 23;10(3):e0121120. doi: 10.1371/journal.pone.0121120 (PMC4370848; doi:10.1371/journal.pone.0121120)

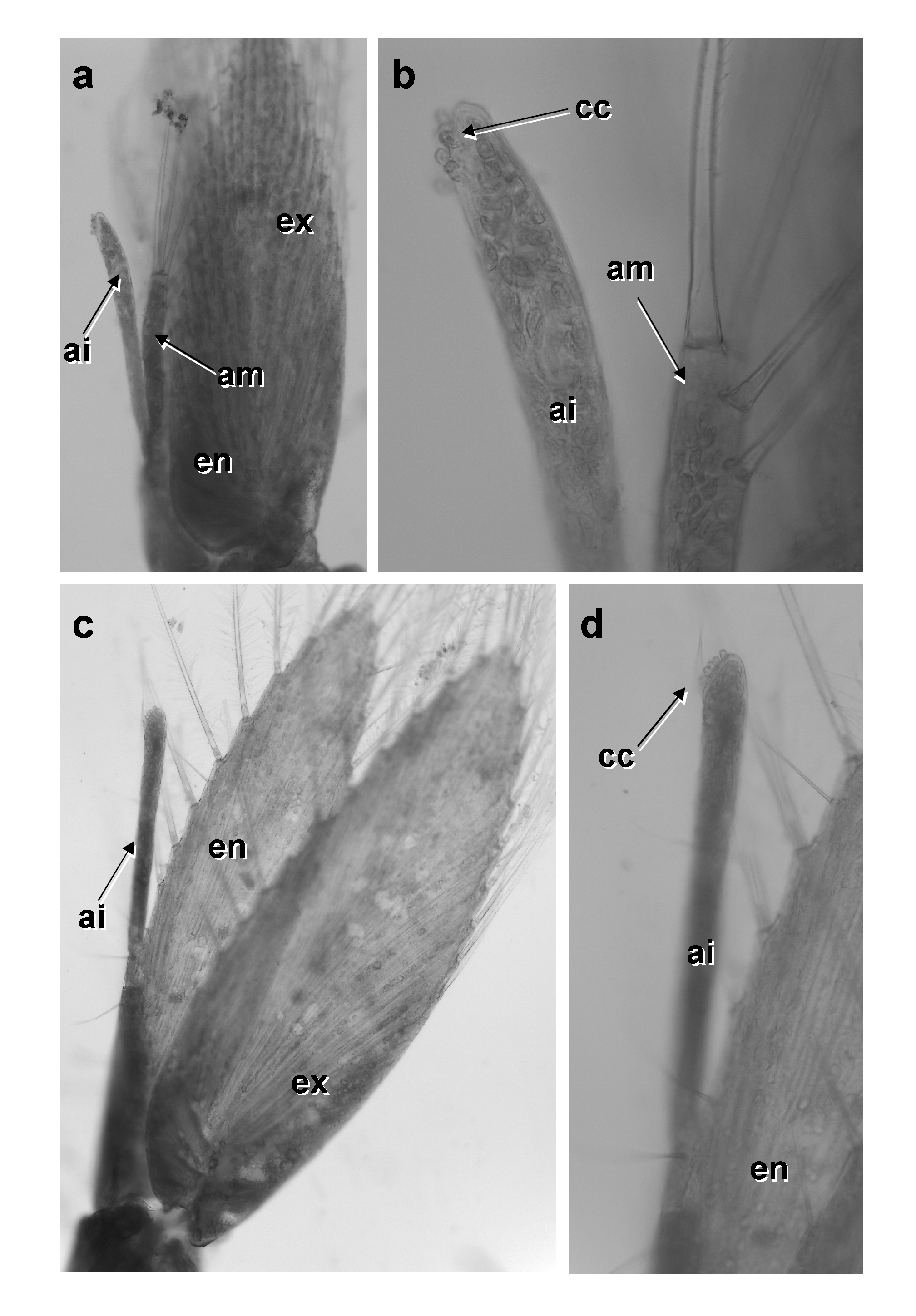

Supplement: S1 Fig — (A) Endopod (en) and exopod (ex) of second pleopod in male individual. The inner margin of the second pleopod in males exhibit an appendix masculina (am) bearing spines and appendix interna (ai). (B) Close-up of the appendix masculina (am) bearing spines and appendix interna (ai) bearing coupling hooks (cc) in males. (C) Endopod (en) and exopod (ex) of second pleopod in female individual. The inner margin of the second pleopod in females exhibit an appendix interna (ai) but lacks an appendix masculina (am). (D) Close-up of the appendix appendix interna (ai) bearing coupling hooks (cc) in females. (TIF) [file pone.0121120.s001.tif]
